# Multifunctional targeting vinorelbine plus tetrandrine liposomes for treating brain glioma along with eliminating glioma stem cells

**DOI:** 10.18632/oncotarget.8360

**Published:** 2016-03-25

**Authors:** Xue-tao Li, Wei Tang, Ying Jiang, Xiao-min Wang, Yan-hong Wang, Lan Cheng, Xian-sheng Meng

**Affiliations:** ^1^ School of Pharmacy, Liaoning University of Traditional Chinese Medicine, Dalian 116600, China; ^2^ Linyi Food and Drug Testing Center, Linyi 276000, China

**Keywords:** multifunctional targeting liposomes, polyethylenimine, vapreotide, glioma stem cells, antitumor efficacy

## Abstract

Malignant brain glioma is the most lethal and aggressive type of cancer. Surgery and radiotherapy cannot eliminate all glioma stem cells (GSCs) and blood–brain barrier (BBB) restricts the movement of antitumor drugs from blood to brain, thus leading to the poor prognosis with high recurrence rate. In the present study, the targeting conjugates of cholesterol polyethylene glycol polyethylenimine (CHOL-PEG_2000_-PEI) and D-a-tocopheryl polyethylene glycol 1000 succinate vapreotide (TPGS_1000_-VAP) were newly synthesized for transporting drugs across the BBB and targeting glioma cells and GSCs. The multifunctional targeting vinorelbine plus tetrandrine liposomes were constructed by modifying the targeting conjugates. The studies were undertaken on BBB model, glioma cells, GSCs, and glioma-bearing mice. *In vitro* results showed that multifunctional targeting drugs-loaded liposomes with suitable physicochemical property could enhance the transport drugs across the BBB, increase the intracellular uptake, inhibit glioma cells and GSCs, penetrate and destruct the GSCs spheroids, and induce apoptosis via activating related apoptotic proteins. *In vivo* results demonstrated that multifunctional targeting drugs-loaded liposomes could significantly accumulate into brain tumor location, show the specificity to tumor sites, and result in a robust overall antitumor efficacy in glioma-bearing mice. These data suggested that the multifunctional targeting vinorelbine plus tetrandrine liposomes could offer a promising strategy for treating brain glioma.

## INTRODUCTION

Malignant glioma is a type of tumor that starts in brain or spine and accounts for 80% of all brain tumors. Nowadays, a combined approach using chemotherapy, surgery and radiotherapy is applied for the treatment of brain glioma. However, there is still a big challenge due to the highly proliferative and infiltrative nature [1]. The median patient survival is only 1–2 years and no significant differences in overall survival are found after the conventional treatment [2]. The therapeutic failure of the combination treatment is related to the following factors: 1) the blood-brain barrier (BBB) provides significant barrier to the effective chemotherapy [3]; 2) the multidrug resistance (MDR) results in incomplete therapeutic response and unsuccessful tumor chemotherapy [4]; 3) the existence of glioma stem cells (GSCs) is responsible for invasion, recurrence and metastasis of brain glioma [5]. Therefore, it is crucial to find an approach to transport drugs effectively across the BBB, inhibit the MDR and then eliminate all the glioma cells and GSCs.

BBB is formed by brain endothelial cells and works as a highly selective permeability barrier. It restricts the passage of solutes, lipophilic drugs, potential neurotoxins and antitumor agents from blood into the central nervous system via an active transport mechanism mediated by P-glycoprotein (P-gp) which is overexpressed on the BBB [6]. Nevertheless, physiological substances, such as water, lipid-soluble molecules, glucose and amino acids, could cross BBB by passive diffusion or selective transport [7]. These phenomena suggest that we could enhance the transport drug carrier across BBB via selecting a suitable mediated endocytosis or blocking the expression of P-gp protein [8, 9]. MDR is defined as the resistance of tumor cells to the cytotoxic effects of many structurally and functionally related or unrelated chemotherapeutic drugs [10]. It leads to the metastasis and relapse of tumor, hence resulting in the failure of clinical treatment [11, 12]. P-gp protein is the mainly MDR-mediated protein and bilaterally transports drugs or substrates across the bilayer: inward uptake or outward efflux [13, 14]. GSCs are now accepted as the “seed” cells of glioma, and are capable of self-renewal, proliferation, differentiation and multidrug resistance [15]. GSCs are proposed to persist in tumors as a distinct population and cause relapse and metastasis by giving rise to new tumors [16]. For the stronger capability in DNA repair and the quiescent situations in cell cycles, GSCs are insensitive and resistant to conventional chemotherapy [17, 18]. Therefore, development of specific therapies targeted to GSCs holds hope for improvement of survival and quality of life of glioma patients.

Polyethylenimine (PEI) is a polymer with repeating unit and possesses high positive charge density due to the presence of ammonium (NH_4_^+^) ions [19]. PEI has been extensively used in the drug delivery system to deliver oligonucleotides, siRNA, and plasmid DNA *in vitro* and *in vivo* [20, 21]. Moreover, the cationic charge centers of targeting ligand modified on the surface of the drug carrier can make it easier to cross the BBB and then target to GSCs via adsorptive-mediated endocytosis [22, 23]. Vapreotide (VAP) is a synthetic somatostatin analog. It is used in the treatment of esophageal variceal bleeding in patients [24]. Some studies have demonstrated VAP could be used as a ligand for targeting drug delivery based on its high affinity to somatostatin receptors which is overexpressed in many tumor cells [25, 26].

Vinorelbine is an anti-mitotic chemotherapy drug and has been used to treat some types of cancers, including breast cancer, non-small cell lung cancer and brain glioma [27]. The agent is the first semi-synthetic vinca alkaloid and the antitumor activity is due to inhibition of mitosis through interaction with tubulin [28, 29]. Tetrandrine (TET) is a bis-benzylisoquinoline alkaloid, and it has been used as a calcium channel blocker. Tetrandrine has anti-inflammatory, immunologic and antiallergenic effects [30]. Recent studies have shown that tetrandrine possesses the potent and specific activity in reversing MDR mediated by P-gp protein which overexpresses on BBB and GSCs [31–33].

In the present study, we hypothesized that a kind of multifunctional targeting vinorelbine plus tetrandrine liposomes modified with PEI and VAP could offer a comprehensive strategy to transport drugs across the BBB, inhibit the MDR, and then eliminate glioma cells and GSCs. In the targeting liposomes, the newly synthesized cholesterol polyethylene glycol polyethylenimine (CHOL-PEG_2000_-PEI) conjugate was modified on the surface of the liposomes and used as a targeting molecule for transporting drugs across the BBB, the D-a-tocopheryl polyethylene glycol 1000 succinate vapreotide (TPGS_1000_-VAP) conjugate was incorporated onto drugs-loaded liposomes and used as a functional molecule for targeting glioma cells and GSCs via receptor-mediated endocytosis, tetrandrine was incorporated into the lipid bilayer for inhibiting the MDR via blocking the expression of P-gp protein which overexpressed on BBB and GSCs, and vinorelbine was entrapped into the liposomal vesicles as antitumor drug. The objectives of this study were to construct the multifunctional targeting drugs-loaded liposomes, and evaluate the antitumor efficacy and the action mechanism of eliminating glioma cells and GSCs.

## RESULTS

### Characterization of the targeting molecules and the liposomes

**Figure 1:**
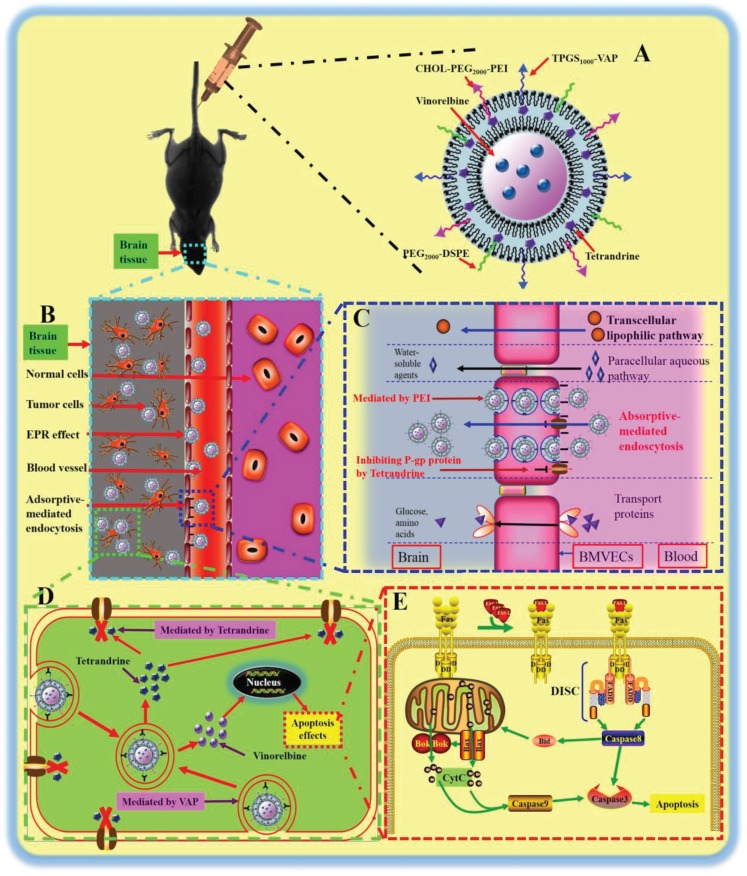
The enhanced antitumor effects of multifunctional targeting vinorelbine plus tetrandrine liposomes Notes: (**A**) A schematic representation of multifunctional targeting vinorelbine plus tetrandrine liposomes. (**B**) A schematic representation of brain site in glioma-bearing mice. (**C**) The enhanced transport drug carrier across the BBB. (**D**) The active targeting and the inhibiting P-gp protein mediated by VAP and tetrandrine, respectively. (**E**) The apoptosis pathway of GSCs.

**Figure 2:**
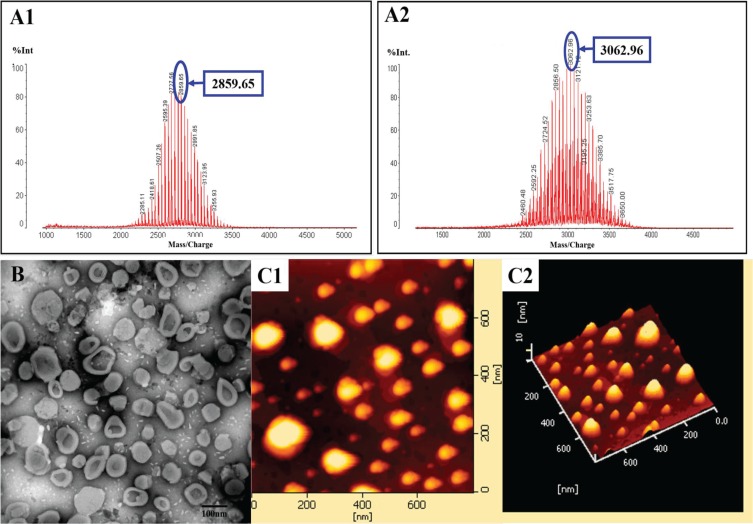
Characterization of targeting molecular materials and liposomes Notes: (**A**) MALDI-TOF-MS spectra of TPGS_1000_-VAP conjugate (**A1**) and CHOL-PEG_2000_-PEI conjugate (**A2**). (**B**) TEM images of multifunctional targeting vinorelbine plus tetrandrine liposomes. (**C**) AFM images of multifunctional targeting vinorelbine plus tetrandrine liposomes (**C1** and**C2**).

Figure 1 shows the enhanced antitumor effects of multifunctional targeting vinorelbine plus tetrandrine liposomes. Figure 2A shows MALDI-TOF-MS spectra of TPGS_1000_-VAP conjugate (A1) and CHOL-PEG_2000_-PEI conjugate (A2). To obtain TPGS_1000_-VAP conjugate, TPGS_1000_-COOH was firstly synthesized, and the activated carboxylic group of TPGS_1000_-COOH was coupled to the amino group of VAP using EDC and NHS as coupling agents. To synthesize CHOL-PEG_2000_-PEI conjugate, PEI was conjugated to distal end of the CHOL-PEG_2000_-NHS by a nucleophilic substitution reaction. The MALDI-TOF-MS spectra exhibited the average masses of TPGS_1000_-VAP conjugate and CHOL-PEG_2000_-PEI conjugate were m/z 2859.65 and m/z 3062.96, respectively. Figure S1 showed that the average masses of TPGS_1000_ and CHOL-PEG_2000_-NHS were m/z 1710.02 and m/z 2463.72, and the average masses of the two targeting molecules were equal to the summation of the raw material masses, indicating the successful synthesis.

Figure 2B and 2C show TEM and AFM morphology of multifunctional targeting vinorelbine plus tetrandrine liposomes, respectively. Results demonstrated that the multifunctional targeting drugs-loaded liposomes were three-dimensional spheroids shaped and displayed a smooth surface with approximately 100 nm in diameter. Table 1 lists the measured particle sizes, polydispersity index (PDI), zeta potential values and encapsulation efficiencies of the varying liposomes. In all liposomal formulations, the average particle sizes were approximately 100 nm with a narrow PDI (≤ 0.20), the encapsulation efficiencies of vinorelbine and tetrandrine were > 90% and > 89%, respectively. The zeta potential values of multifunctional targeting vinorelbine plus tetrandrine liposomes and PEI-modified targeting vinorelbine liposomes were 24.35 ± 4.76 mV and 23.32 ± 3.88 mV which were higher than those of vinorelbine liposomes (−3.33 ± 1.03 mV). For the multifunctional targeting vinorelbine plus tetrandrine liposomes, the *in vitro* release rate of vinorelbine in the simulated body fluids was 28.65 ± 3.65% at 48 h (Figure S2C).

**Table 1:** Characterization of liposomes

	Encapsulation efficiency (%)		Particle size (nm)	PDI	Zeta potential (mV)
	Vinorelbine	Tetrandrine			
Blank multifunctional targeting liposomes	–	–	98.77 ± 1.54	0.186 ± 0.021	−6.25 ± 0.53
Vinorelbine liposomes	93.51 ± 3.54	–	93.55 ± 1.22	0.183 ± 0.012	−3.33 ± 1.03
Vinorelbine plus tetrandrine liposomes	92.26 ± 1.15	90.42 ± 3.55	95.93 ± 2.65	0.193 ± 0.005	−4.38 ± 0.24
VAP-modified targeting vinorelbine liposomes	90.42 ± 1.21	–	100.24 ± 3.64	0.177 ± 0.011	−1.53 ± 0.34
PEI-modified targeting vinorelbine liposomes	95.33 ± 0.75	–	99.75 ± 1.86	0.162 ± 0.017	23.32 ± 3.88
Multifunctional targeting vinorelbine plus tetrandrine liposomes	94.85 ± 1.43	89.453 ± 1.86	102.05 ± 0.99	0.193 ± 0.003	24.35 ± 4.76

### Intracellular uptake and distribution in C6 cells and GSCs

**Figure 3:**
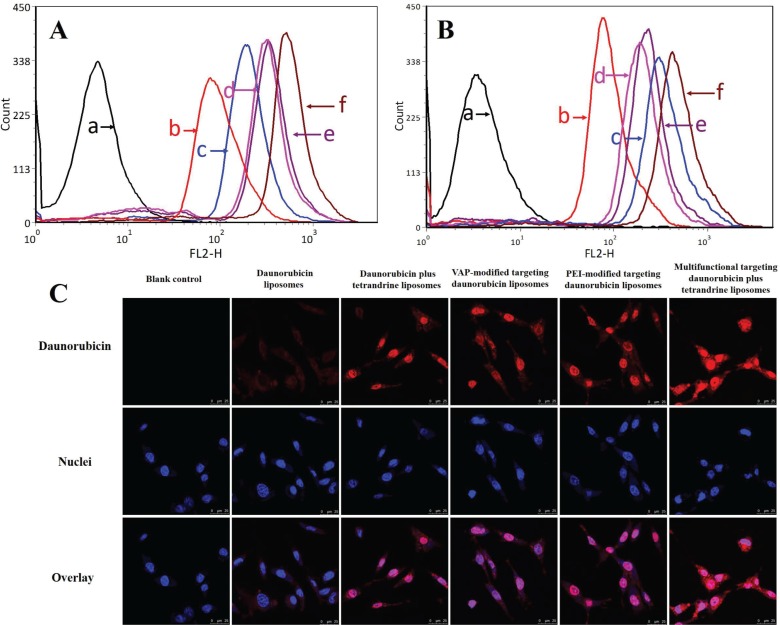
Intracellular uptake in glioma cells and GSCs and distribution in GSCs Notes: (**A**) Intracellular uptake in glioma cells. (**B**) Intracellular uptake in GSCs. (**C**) Confocal images of GSCs treated with varying formulations. a. Blank control; b. Daunorubicin liposomes; c. Daunorubicin plus tetrandrine liposomes; d. VAP-modified targeting daunorubicin liposomes; e. PEI-modified targeting daunorubicin liposomes; f. Multifunctional targeting daunorubicin plus tetrandrine liposomes.

Figure 3A and 3B show the intracellular uptake by C6 cells and GSCs after treatments with varying formulations. The semi-quantitative evaluation by flow cytometry showed that the geometric mean intensity value rank in C6 cells was multifunctional targeting daunorubicin plus tetrandrine liposomes > PEI-modified targeting daunorubicin liposomes > VAP-modified targeting daunorubicin liposomes > daunorubicin plus tetrandrine liposomes > daunorubicin liposomes, and that in GSCs was multifunctional targeting daunorubicin plus tetrandrine liposomes > daunorubicin plus tetrandrine liposomes > PEI-modified targeting daunorubicin liposomes > VAP-modified targeting daunorubicin liposomes > daunorubicin liposomes. For all liposomal formulations, the fluorescent intensity of multifunctional targeting daunorubicin plus tetrandrine liposomes was highest both in C6 cells and GSCs. Figure 3C illustrates the intracellular targeting effects after incubations with the varying formulations. Results showed that multifunctional targeting daunorubicin plus tetrandrine liposomes exhibited higher fluorescence intensity than daunorubicin liposomes did in GSCs, indicating more drugs had been internalized by GSCs.

### Inhibitory effects to C6 cells and GSCs

**Figure 4:**
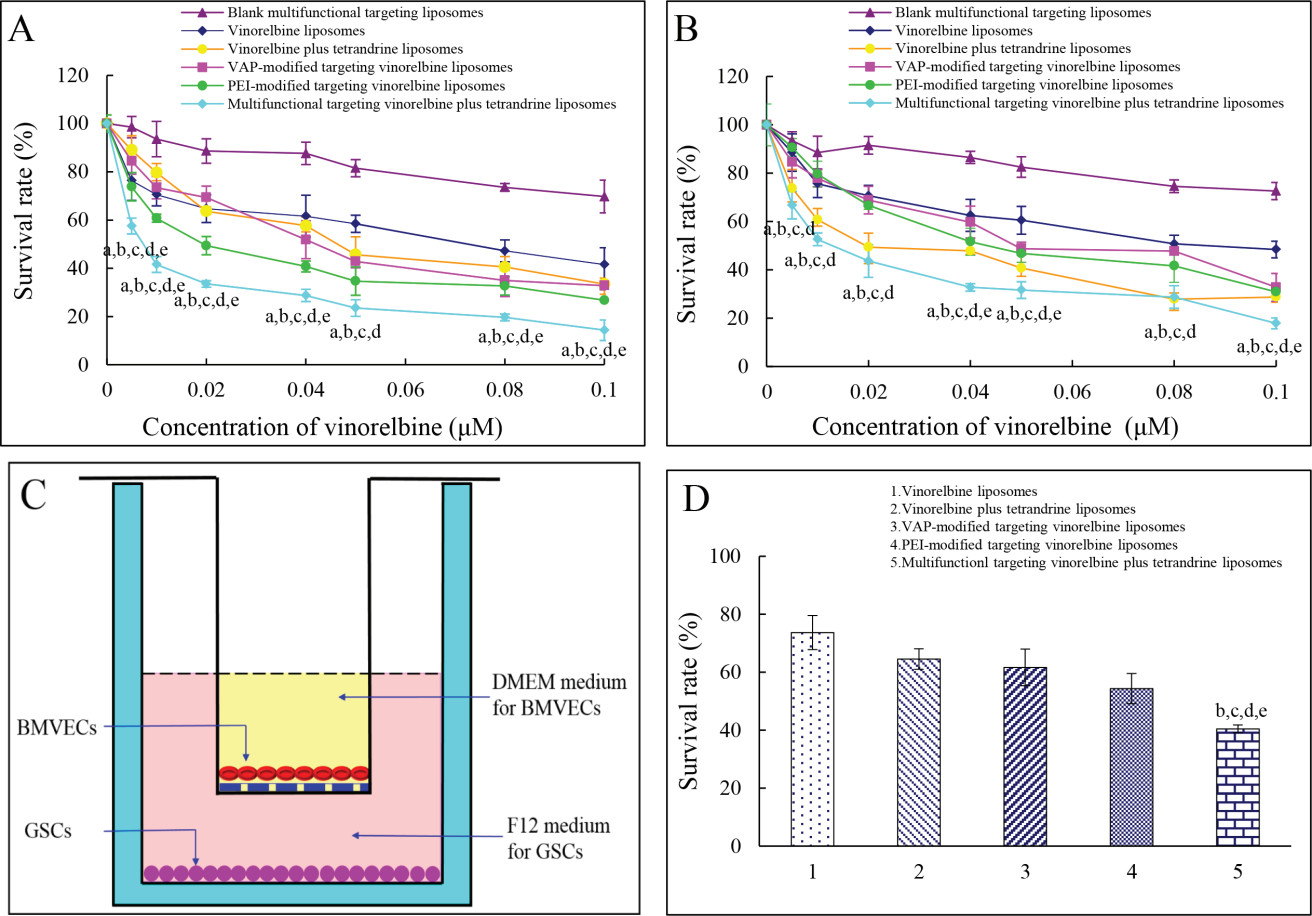
Inhibitory effects to glioma cells and GSCs and the multifunctional targeting effects in BBB model Notes: (**A**) Inhibitory effects to glioma cells; (**B**) Inhibitory effects to GSCs; (**C**) Co-culture BBB model; (**D**) Inhibitory effects to GSCs after crossing the BBB. Data are presented as mean ± SD (*n* = 6). *p* < 0.05; a, vs. blank multifunctional targeting liposomes; b, vs. vinorelbine liposomes; c, vs. vinorelbine plus tetrandrine liposomes; d, vs. VAP-modified targeting vinorelbine liposomes; e, vs. PEI-modified targeting vinorelbine liposomes.

Figure 4A and 4B exhibit the inhibitory effects to C6 cells and GSCs. Results showed that the rank of inhibitory effects to C6 cells was multifunctional targeting vinorelbine plus tetrandrine liposomes > PEI-modified targeting vinorelbine liposomes > VAP-modified targeting vinorelbine liposomes > vinorelbine plus tetrandrine liposomes > vinorelbine liposomes, and that to GSCs was multifunctional targeting vinorelbine plus tetrandrine liposomes > vinorelbine plus tetrandrine liposomes > PEI-modified targeting vinorelbine liposomes > VAP-modified targeting vinorelbine liposomes > vinorelbine liposomes. The results demonstrated that multifunctional targeting vinorelbine plus tetrandrine liposomes exhibited the strongest inhibitory effects to both C6 cells and GSCs at varying dose levels.

### Evaluations on BBB *in vitro*

Figure 4C is the schematic for a co-culture BBB model and Figure 4D is the assay of transport capabilities across BBB. Results showed that the rank of inhibitory effects to GSCs was multifunctional targeting vinorelbine plus tetrandrine liposomes > PEI-modified targeting vinorelbine liposomes > VAP-modified targeting vinorelbine liposomes > vinorelbine plus tetrandrine liposomes > vinorelbine liposomes. The multifunctional targeting vinorelbine plus tetrandrine liposomes exhibited a significant transporting effect across the BBB.

### Induction of apoptotic effects to GSCs

**Figure 5:**
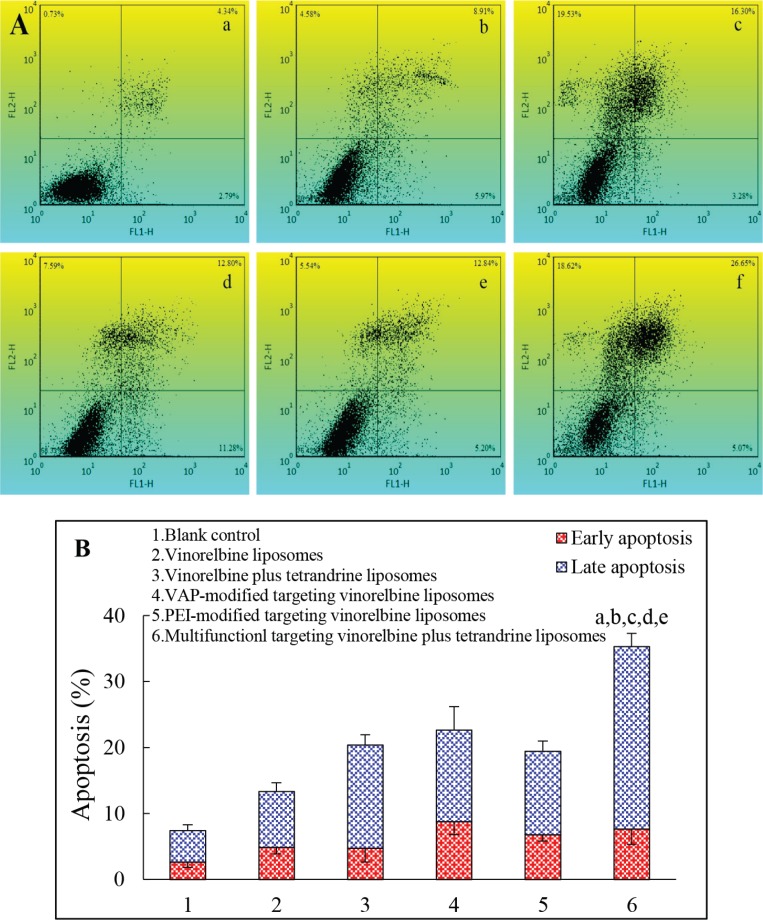
Induced apoptosis in GSCs after treatments with varying formulations Notes: (**A**) Plots by flow cytometry of apoptosis-inducing effects in GSCs; (**B**) Apoptosis in GSCs after treatments with varying formulations. Data are presented as the mean ± SD (*n* = 3). a. Blank control; b. Vinorelbine liposomes; c. Vinorelbine plus tetrandrine liposomes; d. VAP-modified targeting vinorelbine liposomes; e. PEI-modified targeting vinorelbine liposomes; f. Multifunctional targeting vinorelbine plus tetrandrine liposomes. *p* < 0.05; a, vs. blank control; b, vs. vinorelbine liposomes; c, vs. vinorelbine plus tetrandrine liposomes; d, vs. VAP-modified targeting vinorelbine liposome; e, vs. PEI-modified targeting vinorelbine liposomes.

Figure 5 depicts the *in vitro* apoptosis-inducing effects to GSCs. The apoptosis-inducing effects were evaluated by counting the apoptotic percentage during the early period and the late period. After applying the culture medium, vinorelbine liposomes, vinorelbine plus tetrandrine liposomes, VAP-modified targeting vinorelbine liposomes, PEI-modified targeting vinorelbine liposomes and multifunctional targeting vinorelbine plus tetrandrine liposomes, the total percentages of apoptosis in GSCs were 7.41 ± 1.66%, 13.33 ± 2.30%, 20.40 ± 3.65%, 22.64 ± 5.52%, 19.42 ± 2.49% and 35.29 ± 4.34%, respectively. For all liposomal formulations, multifunctional targeting vinorelbine plus tetrandrine liposomes showed the strongest apoptosis-inducing effect to GSCs.

### Regulating apoptotic proteins and P-gp protein

**Figure 6:**
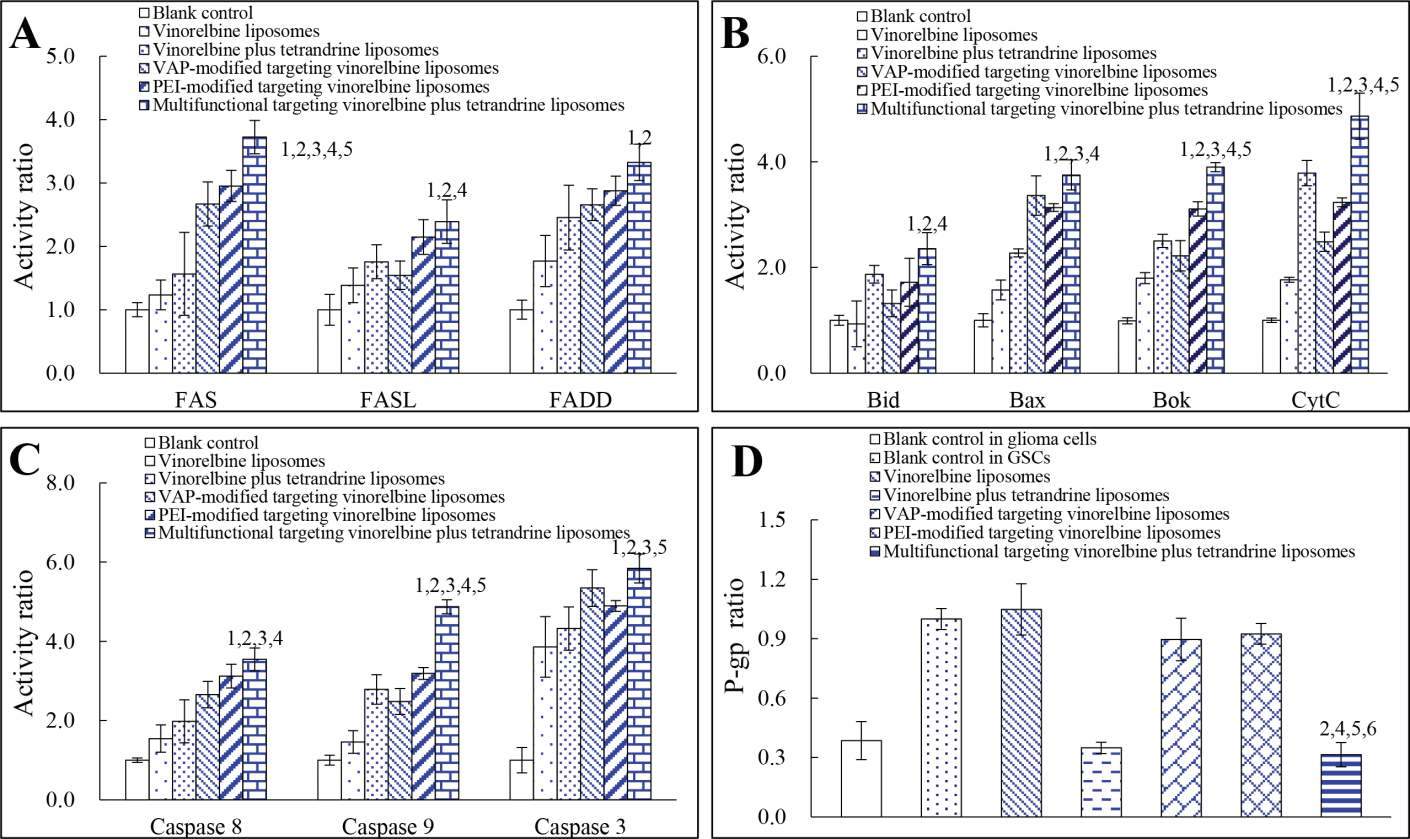
Regulating effects to apoptotic enzymes and P-gp protein in GSCs after treatments with varying formulations Notes: (**A**) Activity of apoptotic receptor/ligand; (**B**) Activity of pro-apoptotic proteins; (**C**) Activity of apoptotic enzymes; (**D**) Activity of P-gp protein. Data are presented as the mean ± SD (*n* = 3). *p* < 0.05; 1, vs. blank control; 2, vs. vinorelbine liposomes; 3, vs. vinorelbine plus tetrandrine liposomes; 4, vs. VAP-modified targeting vinorelbine liposomes; 5, vs. PEI-modified targeting vinorelbine liposomes; 6, vs. Blank control in GSCs.

Figure 6A displays the regulating effects to apoptotic receptor/ligand in GSCs. After treatment with multifunctional targeting vinorelbine plus tetrandrine liposomes for 24 h, the expression ratios of Fas, FasL and FADD in GSCs were 3.72 ± 0.26, 2.39 ± 0.34 and 3.33 ± 0.29 which were evidently higher than those treated with blank control (Fas, FasL or FADD = 1). Figure 6B exhibits the expression levels of apoptotic proteins in GSCs. Compared with control formulations, after treatment with multifunctional targeting vinorelbine plus tetrandrine liposomes, the expression levels of Bid, Bax, Bok and CytC were enhanced to 2.36 ± 0.30, 3.75 ± 0.28, 3.90 ± 0.09 and 4.87 ± 0.44 folds, respectively. Figure 6C illustrates the activities of apoptotic enzymes caspase 8, 9 and 3 in GSCs. After addition of multifunctional targeting vinorelbine plus tetrandrine liposomes for 24 h, the activities of caspase 8, 9 and 3 in GSCs were 3.54 ± 0.29, 4.87 ± 0.18 and 5.84 ± 0.36, respectively. Figure 6D shows the activity of P-gp protein in C6 cells and GSCs before and after treatments with the varying formulations. Before drug treatments, the activity of P-gp protein in C6 cells (0.39 ± 0.10) were evidently lower than those in GSCs (1 ± 0.05). After treatments with vinorelbine liposomes, vinorelbine plus tetrandrine liposomes, VAP-modified targeting vinorelbine liposomes, PEI-modified targeting vinorelbine liposomes and multifunctional targeting vinorelbine plus tetrandrine liposomes for 24 h, the activity ratios of P-gp protein in GSCs were lowered to 1.05 ± 0.13, 0.35 ± 0.03, 0.90 ± 0.11, 0.92 ± 0.05 and 0.31 ± 0.06, respectively.

### Penetration abilities and destruction effects to GSCs spheroids

**Figure 7:**
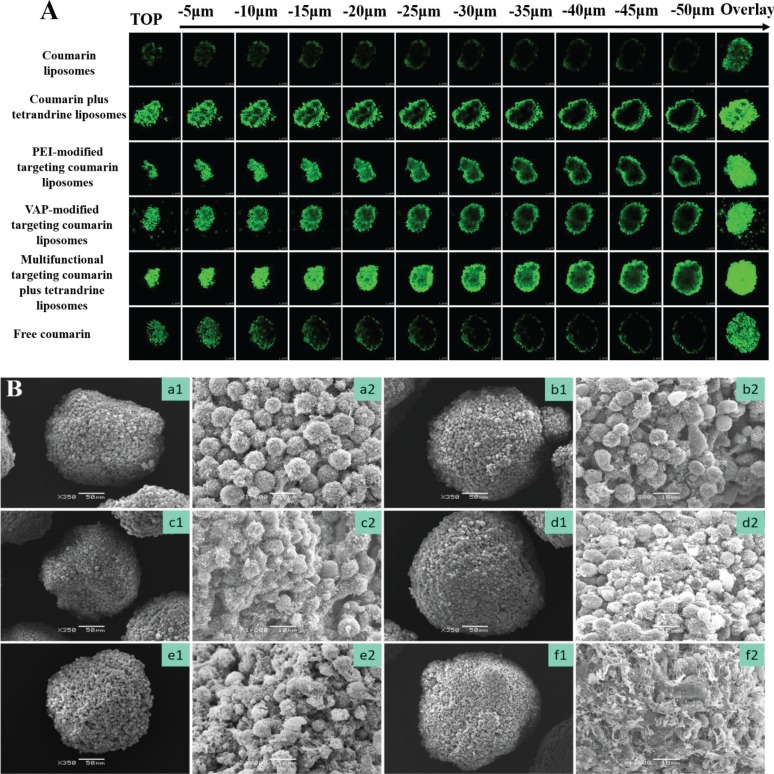
Penetrating abilities and destructing effects to GSCs spheroids Notes: (**A**) Confocal images at different layers from top to middle of the GSCs spheroids; (**B**) SEM photographs of GSCs spheroids. a. Blank control; b. Vinorelbine liposomes; c. Vinorelbine plus tetrandrine liposomes; d. VAP-modified targeting vinorelbine liposomes; e. PEI-modified targeting vinorelbine liposomes; f. Multifunctional targeting vinorelbine plus tetrandrine liposomes. 1, ×350; 2, ×1800.

Figure 7A shows the penetrating ability into GSCs spheroids. Results showed that GSCs spheroids were round-shaped spheres with an average size of 200–300 μm in diameter. For all the varying formulations, the rank of fluorescent intensities in spheroids was multifunctional targeting coumarin plus tetrandrine liposomes > PEI-modified targeting coumarin liposomes ≥ VAP-modified targeting coumarin liposomes ≥ coumarin plus tetrandrine liposomes > free coumarin ≥ coumarin liposomes. Fluorescence overlay of different layers in GSCs spheroids indicated that the multifunctional targeting liposomes had a strong penetrating ability. Figure 7B represents the destruction effects to GSCs spheroids. Results showed that the tightly organized spheroids became disintegrated and shrunken after treatment with multifunctional targeting vinorelbine plus tetrandrine liposomes. For all the liposomal formulations, the rank of destruction effects to the spheroids was multifunctional targeting vinorelbine plus tetrandrine liposomes > vinorelbine plus tetrandrine liposomes ≥ PEI-modified targeting vinorelbine liposomes ≥ VAP-modified targeting vinorelbine liposomes > vinorelbine liposomes.

### *In vivo* imaging and specificity to glioma sites in glioma-bearing mice

**Figure 8:**
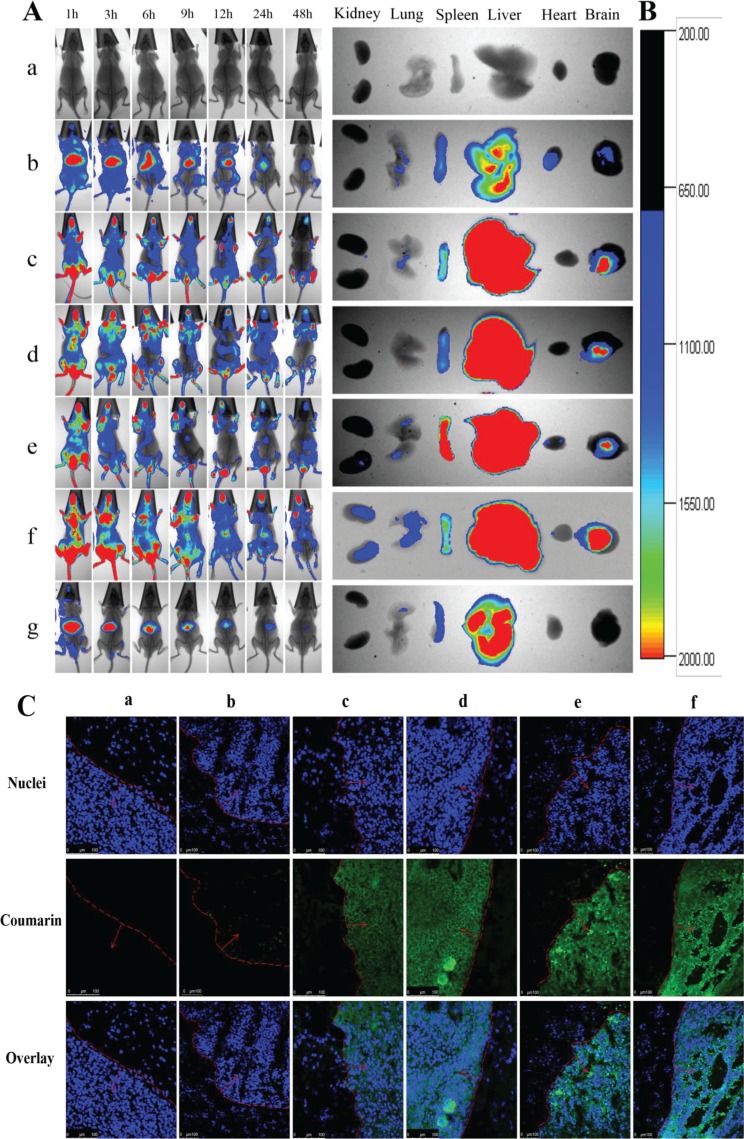
*In vivo* real-time imaging observation and the specificity to glioma sites in glioma-bearing mice Notes: (**A**) *In vivo* real-time images; (**B**) *Ex vivo* optical images of tumor masses and organs at 48 h; (**C**) The specificity to glioma sites. a. Blank control; b. DiR (Coumarin) liposomes; c. DiR (Coumarin) plus tetrandrine liposomes; d. VAP-modified targeting DiR (Coumarin) liposomes; e. PEI-modified targeting DiR (Coumarin) liposomes; f. Multifunctional targeting DiR (Coumarin) plus tetrandrine liposomes; g. Free DiR.

Figure 8A depicts the real-time distribution and accumulation ability of the varying formulations in glioma-bearing mice inoculated with GSCs. After intravenous injection of multifunctional targeting DiR plus tetrandrine liposomes, a strong fluorescent signal was observed throughout the entire blood circulatory system and the tumor tissue at the early stage, and the fluorescent signal was maintained up to 48 h in tumor location. In contrast, after administration of free DiR fluorescent dye, the fluorescent signal was rapidly distributed in the liver and gradually weakened or disappeared after 24 h. For all liposomal formulations, the rank of DiR fluorescent signal in brain tumor masses at designated time-point was multifunctional targeting DiR plus tetrandrine liposomes > DiR plus tetrandrine liposomes ≥ VAP-modified targeting DiR liposomes ≥ PEI-modified targeting DiR liposomes > DiR liposomes > free DiR. Figure 8B illustrates the *ex vivo* optical images of brain tumor masses and major organs after the glioma-bearing mice were sacrificed at 48 h. Results exhibited that the fluorescence of the multifunctional targeting DiR plus tetrandrine liposomes was still observed in brain tumor masses and in major organs (liver and spleen). In contrast, the fluorescence signal was weakly visible in the liver and spleen, and was invisible in tumor masses after injection of free DiR. Figure 8C depicts the specificity to glioma sites after treatments with varying formulations. Results showed that a strong green fluorescent signal was observed in tumor sites after treatment with multifunctional targeting coumarin plus tetrandrine liposomes. In contrast, the fluorescent signal was too weak to be distinguished in tumor sites after administration of free coumarin.

### Antitumor efficacy in glioma-bearing mice

**Figure 9:**
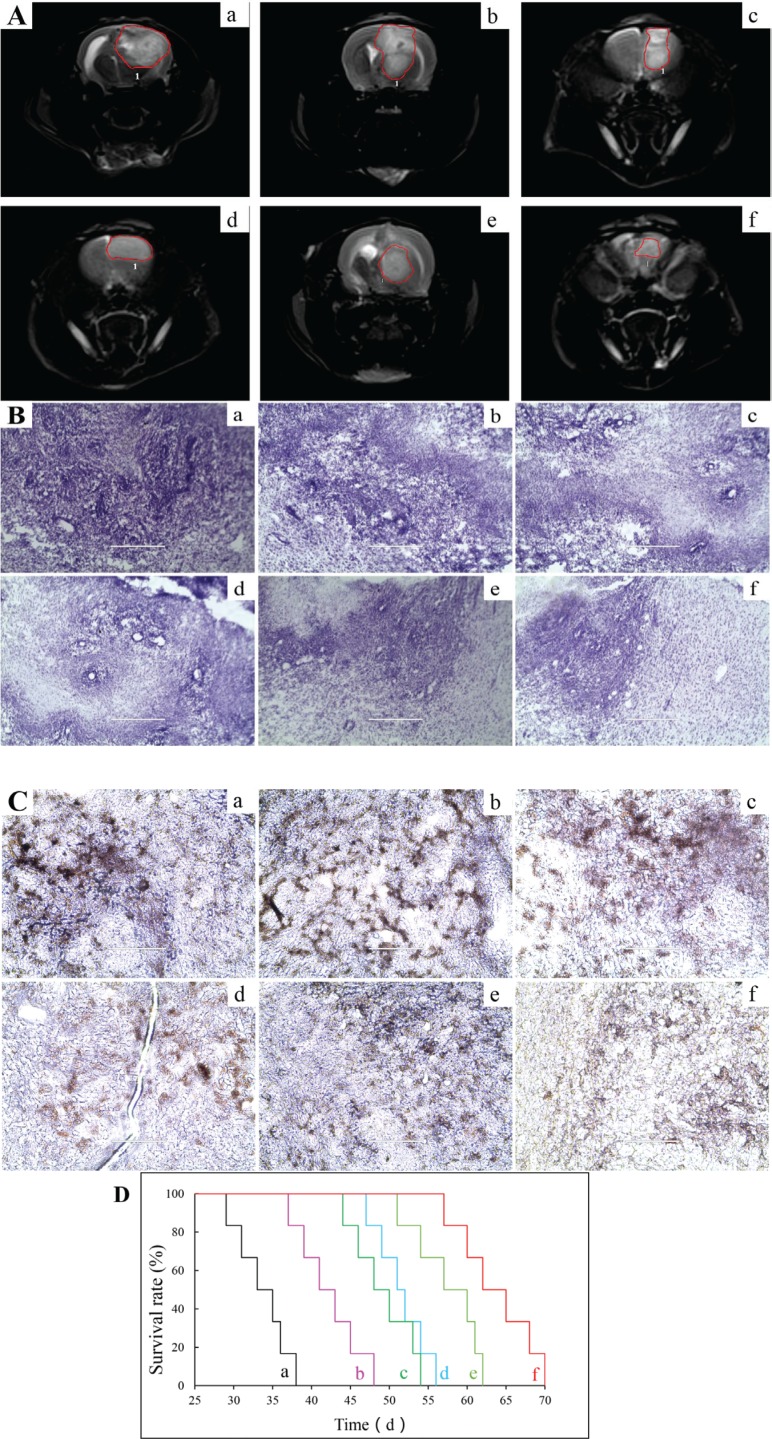
The antitumor efficacy in glioma-bearing mice after treatments with varying formulations Notes: (**A**) MRI images of brain tissues; (**B**) Nissl staining of tumor masses; (**C**) Immunohistochemical staining for CD133 on the brain glioma sites; (**D**) Kaplan-Meier survival curves. a. Blank control; b. Vinorelbine liposomes; c. Vinorelbine plus tetrandrine liposomes; d. VAP-modified targeting vinorelbine liposomes; e. PEI-modified targeting vinorelbine liposomes; f. Multifunctional targeting vinorelbine plus tetrandrine liposomes.

Figure 9A shows the MRI image of brain tissues in glioma-bearing mice. The rank of tumor diameter in brain was physiological saline > vinorelbine liposomes > vinorelbine plus tetrandrine liposomes ≥ VAP-modified targeting vinorelbine liposomes ≥ PEI-modified targeting vinorelbine liposomes > multifunctional targeting vinorelbine plus tetrandrine liposomes. Figure 9B illustrates Nissl staining of tumor masses after treatments with the varying formulations. Results demonstrated that the abnormal cells in tumor sites were diminished after treatment with multifunctional targeting vinorelbine plus tetrandrine liposomes as compared with those treated with physiological saline. Figure 9C depicts the immunohistochemical staining for CD133 on the brain glioma sites after treatments with the varying formulations. In terms of the number of GSCs, the rank of the groups was physiological saline > vinorelbine liposomes > vinorelbine plus tetrandrine liposomes ≥ VAP-modified targeting vinorelbine liposomes ≥ PEI-modified targeting vinorelbine liposomes > multifunctional targeting vinorelbine plus tetrandrine liposomes. Figure 9D represents Kaplan-Meier survival curves of glioma-bearing mice arising from GSCs. Results demonstrated that the multifunctional targeting drugs-loaded liposomes have a strong potential for treating glioma-bearing mice. After treatments with saline, free vinorelbine, vinorelbine liposomes, vinorelbine plus tetrandrine liposomes, VAP-modified targeting vinorelbine liposomes, PEI-modified targeting vinorelbine liposomes and multifunctional targeting vinorelbine plus tetrandrine liposomes, the survival ranges were 29–38, 32–41, 37–48, 47–56, 44–54, 52–62 and 57–70 days, respectively. The median survival time of mice treated with multifunctional targeting vinorelbine plus tetrandrine liposomes (63.67 ± 4.93 days) was significantly longer than that treated with saline (33.67 ± 3.33 days). Table S1 shows the blood examination of the glioma-bearing mice after administrations of varying formulations. The results clearly indicated that multifunctional targeting vinorelbine plus tetrandrine liposomes provided the optimal therapeutic efficacy with few side effects.

## DISCUSSION

The regular chemotherapy drugs cannot penetrate into CNS for the obstacle of the BBB and cannot eradicate all the glioma cells and GSCs, thus leading to high recurrence rate [34]. Therefore, in the present study, a kind of nanostructured multifunctional targeting vinorelbine plus tetrandrine liposomes are developed to deliver the drugs across BBB, and then eliminating all the glioma cells and GSCs. The new TPGS_1000_-VAP conjugate and CHOL-PEG_2000_-PEI conjugate were successfully synthesized and confirmed by MALDI-TOF-MS (Figure 2A1 and 2A2). The multifunctional targeting vinorelbine plus tetrandrine liposomes possessed of the following physicochemical characteristics: small and well-distributed particle size, positive potential (Figure S2A and S2B), high encapsulation efficiency (Table 1), smooth surface (Figure 2B and 2C) and delayed drug release (Figure S2C). The optimal particle size, high encapsulation efficiency and delayed drug release enable the multifunctional targeting drugs-loaded liposomes to accumulate into tumor site by the enhanced permeability retention (EPR) effect [35]. The positive charge of PEI will be beneficial for crossing BBB and targeting GSCs via adsorptive-mediated endocytosis.

GSCs were cultured in serum-free medium, and they were proven to have stem-like characteristics with high level of nestin (marked as nestin^+^, 93.39%) as compared with isotype control (Figure S3). The targeting effects of multifunctional targeting drugs-loaded liposomes were evidenced by flow cytometry (Figure 3A and 3B) and confocal observations (Figure 3C). Results demonstrated that multifunctional targeting drugs-loaded liposomes showed a significantly enhanced intracellular uptake in both glioma cells and GSCs as compared with regular liposomes. To obtain direct and visible evidence, daunorubicin was used as a fluoresce probe and the confocal microscope was used to observe the intracellular distribution of daunorubicin in GSCs. Results showed that a strong red fluorescent signal could be clearly observed in GSCs after incubation with multifunctional targeting drugs-loaded liposomes, indicating more drugs had been internalized by GSCs. The mechanism for the enhanced intracellular uptake could be explained by the addition of VAP and PEI, which were modified onto liposomal surface and displayed high binding specificity with the membrane of the GSCs.

In the cytotoxic assays, multifunctional targeting vinorelbine plus tetrandrine liposomes showed the strongest inhibitory effects to both glioma cells (Figure 4A) and GSCs (Figure 4B). This enhanced inhibitory efficacy could be attributed to the increased intracellular uptake of the multifunctional targeting drugs-loaded liposomes by glioma cells or GSCs. In addition, the blank multifunctional targeting liposomes showed slight inhibitory effects to glioma cells and GSCs, indicating that there was a slight toxicity with the multifunctional targeting liposomes themselves.

Transport capabilities across BBB were confirmed in this study using a co-culture BBB model. The TEER values of the BBB model were 273–296 Ω·cm^2^, and they were monitored during the whole process of the experiments to make sure there was no leakage in BBB model. Results indicated that multifunctional targeting vinorelbine plus tetrandrine liposomes evidently improved the transport drugs across the BBB. These outcomes could be explained for the adsorptive-mediated endocytosis and the inhibition of P-gp protein mediated by PEI and tetrandrine (Figure 1C).

Apoptosis is the process of programmed cell death (PCD) that may occur in multicellular organisms and confers advantages during an organism's lifecycle [36]. Results demonstrated that multifunctional targeting drugs-loaded liposomes had the most significant apoptosis-inducing effects to GSCs. The enhanced apoptosis-inducing effects to GSCs could be explained by the following facts: Fas ligand (FasL) trimer causes trimerization of Fas receptor, and then the death-inducing signaling complex (DISC) is formed by the interactions of Fas and FasL. Upon ensuing death domain (DD) aggregation, DISC is internalized via the cellular endosomal machinery, and this allows Fas-associated death domain (FADD) to bind the DD of Fas [37]. After the interactions of FADD and FADD-like ICE, caspase 8 self-activate through proteolytic cleavage. The activated caspase 8 catalyzes the cleavage of the BH3-only protein Bid into its truncated form and increases the activities of pro-apoptotic proteins (Bok and Bax), and then cytochrome c (CytC) is detached from the mitochondrial inner membrane and extruded into the soluble cytoplasm. The release of CytC in turn activates caspase 9. In case of activating the apoptotic enzymes (caspase 8, 9), the down-stream effector (caspase 3) is activated, thus leading to DNA degradation, membrane blebbing, and other hallmarks of apoptosis (Figure 1E) [38].

P-gp protein is an ATP-dependent drug efflux pump for xenobiotic compounds with broad substrate specificity. It is responsible for decreased drug accumulation in multidrug-resistant cells and often mediates the development of resistance to antitumor drugs [39]. In this study, the activity ratios of P-gp protein in C6 cells (0.39 ± 0.10) before drug treatment were distinctly different from those in GSCs (1 ± 0.05) which displayed the resistance to antitumor drugs in GSCs. Results demonstrated that the expression ratios of P-gp protein were dramatically down-regulated after treatments with vinorelbine plus tetrandrine liposomes and multifunctional targeting vinorelbine plus tetrandrine liposomes. The down-regulation indicated that tetrandrine inserted into the liposomal bilayer could circumvent MDR via inhibiting the expression of P-gp protein.

To demonstrate penetrating ability and destructing effects, the varying drugs-loaded liposomes were applied to GSCs spheroids. Results showed that the strongest fluorescent intensity at different layers of the spheroids from top to middle could be observed after treatment with multifunctional targeting coumarin plus tetrandrine liposomes and the fluorescence overlay of different layers in GSCs spheroids displayed the same penetrating ability. In addition, multifunctional targeting vinorelbine plus tetrandrine liposomes showed the most significant destructing effects to GSCs spheroids as compared with other formulations, and the tightly organized spheroids after treated with multifunctional targeting liposomes became disintegrated and shrunken (Figure 7B).

The real-time imaging was performed to understand the distribution status of multifunctional targeting drugs-loaded liposomes in glioma-bearing mice. The DiR was used as a fluorescent probe [40]. According to Figure 8A, multifunctional targeting DiR plus tetrandrine liposomes exhibited the highest accumulation in brain tumor sites up to 48 h and showed a long-lasting stability in blood circulation. The *ex vivo* fluorescent images of major organs further confirmed the above distributions of the multifunctional targeting liposomes (Figure 8B). The specificity to brain glioma sites after treatments with varying formulations was evaluated by a confocal method. Results showed that the strongest green fluorescence was founded in glioma sites after treatment with multifunctional targeting coumarin plus tetrandrine liposomes (Figure 8C), and this could be explained by the enhanced transport drugs across BBB and the increased intracellular uptake mediated by tetrandrine, PEI and VAP.

Antitumor efficacy was evaluated on glioma-bearing mice from several aspects: MRI image, Nissl staining, immunohistochemical staining and survival curves. Results demonstrated that the multifunctional targeting drugs-loaded liposomes had a strong potential for treating glioma-bearing mice (Figure 9). The enhanced antitumor efficacy could be explained by the following aspects: (i) the long circulation in the blood system coming from PEGylated lipid materials on the liposomal vesicles; (ii) the enhanced transport drug across BBB via the adsorptive-mediated endocytosis by PEI and via blocking the expression of P-gp protein by tetrandrine; (iii) the increased intracellular uptake by glioma cells and GSCs via the receptor-mediated endocytosis by VAP.

## CONCLUSIONS

In the present study, the targeting conjugates of CHOL-PEG_2000_-PEI and TPGS_1000_-VAP were newly synthesized, showing the strong transport ability across BBB and the obvious intracellular uptake ability by glioma cells and GSCs. The multifunctional targeting vinorelbine plus tetrandrine liposomes were characterized with suitablephysicochemical property and demonstrated a strong antitumor efficacy both *in vitro* and *in vivo*. The action mechanisms for the enhanced efficacy in treating brain glioma involves the following aspects: (i) PEGylated liposomes prolong the circulation in the blood via avoiding the rapid uptake by reticuloendothelial system (RES) and increase accumulation in tumor sites by EPR effect; (ii) PEI conjugated on the liposomal surface increases the transport drugs across BBB via adsorptive-mediated endocytosis; (iii) VAP incorporated onto drugs-loaded liposomes enhances the intracellular uptake by glioma cells and GSCs via receptor-mediated endocytosis; (iv) Tetrandrine inserted into the liposomal bilayer promotes multifunctional targeting liposomes to cross BBB and improves intracellular uptake by glioma cells and GSCs via inhibiting P-gp protein overexpressed on BBB and GSCs; (v) The multifunctional targeting liposomes induce apoptosis of GSCs via activating apoptotic receptor/ligand (Fas and FasL), up-regulating Fas-associated protein with Death Domain (FADD), activating pro-apoptotic proteins (Bid, Bax and Bok), activating CytC and activating apoptotic enzymes (caspase 8, 9 and 3). Consequently, the above results strongly support our hypothesis (Figure 1), and the multifunctional targeting vinorelbine plus tetrandrine liposomes could provide a useful strategy for eliminating glioma cells and GSCs.

## MATERIALS AND METHODS

### Reagents and cell lines

Vinorelbine tartrate and tetrandrine were purchased from Meilun Biotechnology Co., Ltd. (Dalian, China). PEI (689 Da), 1-(3-Dimethylaminopropyl)-3-ethylcarbodiimide (EDC), N-hydroxysuccinimide (NHS), D-a-tocopheryl polyethylene glycol 1000 succinate (TPGS_1000_), dicyclohexylcarbodiimide (DCC), 4-dimethylamiopryidine (DMAP) and dimethylformamide (DMF) were purchased from Sigma-Aldrich (St. Louis, MO, USA). VAP was synthesized by Shanghai Apeptide Co., Ltd. (Shanghai, China). Dipalmitoyl Phosphatidylcholine (DPPC) was supplied by Nippon Fine Chemical Co., Ltd. (Osaka, Japan). Cholesterol polyethylene glycol NHS (CHOL-PEG_2000_-NHS) was purchased from Nanocs Inc. (NY, USA) and polyethylene glycol -distearoylphosphatidylethanolamine (PEG_2000_-DSPE) was purchased from Avanti Polar Lipids, Inc. (Alabaster, AL, USA). Other chemicals were analytical or chromatographic grade. C6 cells were maintained in Ham's F10 medium (Macgene Biotech Co. Ltd., Beijing, China) supplemented with 10% fetal bovine serum (FBS) (Gibco, Billings, MT, USA). GSCs were grown in DMEM-F12 (Macgene Biotech) supplemented with 2% B27 (Gibco), 20 μg/mL basic fibroblast growth factor and 20 μg/mL epidermal growth factor (Macgene Biotech). Murine brain microvascular endothelial cells (BMVECs) were cultured in the endothelial cell culture medium (DMEM, 20% FBS, 100 U/mL penicillin, 100 mg/mL streptomycin, 2 mmol/L L-glutamine, 100 mg/mL endothelial cell growth factor, 40 U/mL heparin).

### Synthesis of targeting conjugates

TPGS_1000_-VAP conjugate was synthesized according to our earlier researches [22]. Briefly, TPGS_1000_, glutaric acid, DMAP and DCC were dissolved in DMSO (1:5:2.5:6, mol/mol), and the reaction mixture was stirred for 24 h at room temperature. The crude product was dialyzed against deionized water in a dialysis tubing (cut-off MW, 1500) for 48 h. The resultant TPGS_1000_-COOH was lyophilized for 48 h. Afterwards, TPGS_1000_-COOH, VAP, EDC and NHS (1:1:4:6, mol/mol) were dissolved in pyridine-DMSO (1:1), and the reaction mixture was stirred for 12 h at room temperature. The crude product was dialyzed in a dialysis tubing (cut-off MW, 1500) for 48 h. Finally, TPGS_1000_-VAP conjugate was obtained by freeze-drying and stored at −20°C.

CHOL-PEG_2000_-PEI conjugate was synthesized from CHOL-PEG_2000_-NHS and PEI according to our previous reports [41]. Briefly, PEI and CHOL-PEG_2000_-NHS (5:1, mol/mol) were dissolved in anhydrous DMF, and pH value of the reaction solution was adjusted to 9.0 using N-methyl morphine. After being continuously stirred at room temperature for 36 h, the crude product was then transferred to a dialysis tubing (cut-off MW, 2000 Da) and dialyzed against deionized water for 36 h to remove uncoupled PEI, DMF and N-methyl morphine. The resultant was then lyophilized and stored at −20°C. Finally, the conjugates of CHOL-PEG_2000_-PEI and TPGS_1000_-VAP were identified by matrix-assisted laser desorption/ionization time of flight mass spectrometry (MALDI-TOF-MS instrument, Shimadzu, Japan).

### Preparation and characterization of the liposomes

Blank multifunctional targeting liposomes were prepared using the film dispersion method [42]. Briefly, DPPC, cholesterol, PEG_2000_-DSPE, CHOL-PEG_2000_-PEI, TPGS_1000_-VAP and tetrandrine (60:35:3.5:2:2:5, molar ratio) were dissolved in chloroform and methanol (1:1, v/v) in a pear-shaped flask. The solvent was removed by a rotary evaporator, and the lipid film was hydrated with 250 mM ammonium sulfate by sonication in a water bath for 10 min. Subsequently, the suspensions were treated by a probe-type sonicator for another 10 min (200 W). The suspensions were extruded through polycarbonate membranes with the pore size of 200 nm for 3 times, thus producing the blank liposomes. To prepare the drugs-loaded liposomes, the blank liposomes were further dialyzed (cut-off MW, 12,000–14,000) in PBS solutions for 24 h, and then incubated with a certain amount of vinorelbine tartrate in water bath at 40°C with intermittently shaken for 20 min (lipids: drug = 15:1, w/w). The multifunctional targeting vinorelbine plus tetrandrine liposomes were then obtained.

VAP-modified targeting vinorelbine liposomes, PEI-modified targeting vinorelbine liposomes, vinorelbine plus tetrandrine liposomes and vinorelbine liposomes were similarly prepared by excluding the addition of CHOL-PEG_2000_-PEI and tetrandrine, TPGS_1000_-VAP and tetrandrine, CHOL-PEG_2000_-PEI and TPGS_1000_-VAP, tetrandrine and the two targeting conjugates, respectively. Besides, the varying coumarin liposomes (lipids: coumarin = 200:1, w/w), DiR liposomes (lipids: DiR = 300:1, w/w) and daunorubicin liposomes (lipids: drug = 20:1, w/w) were similarly prepared as the fluorescent probes for evaluating targeting effects [43].

Particle size, polydispersity index (PDI) and zeta potential value were measured using a Nano Series Zen 4003 Zetasizer (Malvern Instruments Ltd., Malvern, UK). Atomic force microscopy (AFM; NSK Ltd., Tokyo, Japan) and transmission electron microscopy (TEM; FEI Co., Japan) were used to observe the morphology of the liposomes. Both vinorelbine and tetrandrine were measured by HPLC system with UV detector (Agilent Technologies Inc., CA, USA). Encapsulation efficiency (EE) of vinorelbine or tetrandrine was calculated using the formula: EE = (W_encap_/W_total_) × 100%, where W_total_ and W_encap_ are the measured amounts of drugs in the liposomal suspensions before and after passing over the Sephadex G-100 column, respectively. *In vitro* release of vinorelbine from the varying liposomes was performed by the dialysis against the release medium (PBS containing 10% FBS). Vinorelbine content in the sample was measured by HPLC, and release rate of vinorelbine was estimated as our previous reports [44]. Each assay was repeated in triplicate.

### Intracellular uptake and distribution in C6 cells and GSCs

GSCs were cultured in serum-free medium and identified according to our previous reports [45]. Daunorubicin was used as the fluorescent probe for observing the targeting effects. To study the intracellular uptake, C6 cells or single cell suspensions of GSCs were seeded into 6-well culture plates (4 × 10^5^ cells/well) and incubated for 24 h, and then all the cells were incubated in the medium containing daunorubicin liposomes, daunorubicin plus tetrandrine liposomes, VAP-modified targeting daunorubicin liposomes, PEI-modified targeting daunorubicin liposomes and multifunctional targeting daunorubicin plus tetrandrine liposomes at a concentration of 10 μM daunorubicin. Culture medium was used as blank control. After incubation for 3 h, the cells were washed 3 times with cold PBS, trypsinized and harvested in 300 μL PBS. Fluorescence intensity of daunorubicin was measured using a FACScan flow cytometer (BD Biosciences, NJ, USA) with 1 × 10^4^ cells collected. Each assay was repeated in triplicate.

To observe intracellular distribution visually, GSCs were seeded into chambered coverslips (2 × 10^5^ cells/well). After incubation for 24 h, varying drug formulations were applied into the dishes at a concentration of 10 μM daunorubicin and incubated for another 3 h. Control group was performed with blank medium. The cells were washed 3 times with cold PBS, fixed by 4% paraformaldehyde for 10 min and stained with Hoechst 33258 (2 μg/mL) for 5 min. Finally, the samples were imaged using a confocal laser scanning fluorescent microscopy (Leica, Heidelberg, Germany).

### Cytotoxic effects on C6 cells and GSCs

C6 cells and single GSCs suspensions were seeded into 96-well culture plates at 5 × 10^3^ cells/well and cultured for 24 h under an atmosphere of 5% CO_2_. The medium was then replaced with fresh medium containing varying concentrations of blank multifunctional targeting liposomes, vinorelbine liposomes, vinorelbine plus tetrandrine liposomes, VAP-modified targeting vinorelbine liposomes, PEI-modified targeting vinorelbine liposomes and multifunctional targeting vinorelbine plus tetrandrine liposomes. The final concentration of vinorelbine was in the 0–0.1 μM range. Culture medium was used as blank control. At 48 h, the cytotoxicity was determined using a sulforhodamine B (SRB) staining assay, and the absorbance was read at 540 nm using a microplate reader (Tecan Infinite F50, Shanghai, China). The survival rates were calculated using the following formula: survival % = (A_540 nm_ for the treated cells/A_540 nm_ for the controlled cells) × 100%, where A_540 nm_ is the absorbance value. Finally, dose-effect curves were plotted [46]. Each assay was repeated in triplicate.

### Evaluations on BBB *in vitro*

To understand the potential for transporting drug carrier across BBB, a BMVECs/GSCs co-culture BBB model was established according to the previous reports [47]. Briefly, BMVECs were seeded on the upper side of the insert at a density of 3.5 × 10^4^ cells/insert (Corning, NY, USA; 0.4 μm pore size, 12 mm diameter, and 1.12 cm^2^ surface area). GSCs cells were seeded (2000 cells/well) on the transwell culture plates. The culture medium were changed once every 2 days. After incubation for 6 days, the tightness of BBB model was assessed by measuring the transendothelial electrical resistance (TEER) values. When TEER values were higher than 250 Ω·cm^2^, varying drug formulations were added into the apical inserts of transwells, including vinorelbine liposomes, vinorelbine plus tetrandrine liposomes, VAP-modified targeting vinorelbine liposomes, PEI-modified targeting vinorelbine liposomes and multifunctional targeting vinorelbine plus tetrandrine liposomes. The final concentration of vinorelbine was 2 μM. After incubation for 2 h, the inserts were moved away, and GSCs were further incubated for 48 h, the percentage of surviving GSCs in the basolateral compartment was determined by the SRB assay.

### Induction of apoptotic effects to GSCs

The apoptotic effects to GCSs were detected using a fluorescein isothiocyanate annexin V staining kit (KeyGen Biotechnology Co., Ltd., China). Briefly, single cell suspensions of GSCs were seeded into 6-well culture plates (5 × 10^5^ cells/well) and cultured for 24 h. After incubation for 24 h, cells were treated with vinorelbine liposomes, vinorelbine plus tetrandrine liposomes, VAP-modified targeting vinorelbine liposomes, PEI-modified targeting vinorelbine liposomes and multifunctional targeting vinorelbine plus tetrandrine liposomes at the concentration of 0.5 μM vinorelbine. Blank culture medium was used as control group. After incubation, cells were assessed by a FACScan flow cytometer according to manufacturer instructions [48]. Each assay was repeated in triplicate.

### Regulating apoptotic proteins and P-gp protein

The expression of apoptotic proteins in GSCs was performed using an ELISA kit (Cusabio, Wuhan, China). Briefly, the cells were treated with vinorelbine liposomes, vinorelbine plus tetrandrine liposomes, VAP-modified targeting vinorelbine liposomes, PEI-modified targeting vinorelbine liposomes and multifunctional targeting vinorelbine plus tetrandrine liposomes, respectively. Control experiment was performed by adding culture medium. The final concentration of vinorelbine was 0.5 μM. After incubation for 12 h, cells were harvested and lysed. The total proteins were measured using the bicinchoninic acid (BCA) kit at 540 nm. Cell lysates were analyzed by a microplate reader according to manufacturer instructions of the kits. Each assay was repeated in triplicate.

To evaluate the expression of P-gp protein, C6 cells and GSCs were cultured for 24 h. Then C6 cells were treated with culture medium, and GSCs were treated with culture medium, vinorelbine liposomes, vinorelbine plus tetrandrine liposomes, VAP-modified targeting vinorelbine liposomes, PEI-modified targeting vinorelbine liposomes and multifunctional targeting vinorelbine plus tetrandrine liposomes at a concentration of 0.5 μM vinorelbine, respectively. The cells were further cultured for 12 h. The activity of P-gp protein was analyzed by microplate reader according to manufacturer instruction of the kit [49]. Each assay was repeated in triplicate.

### Penetration ability and destruction effects to GSCs spheroids

To evaluate the penetration ability, coumarin was used as a fluorescent probe. Briefly, after continuous cultured in serum-free medium for 3 weeks, GSCs spheroids were treated with free coumarin, coumarin liposomes, coumarin plus tetrandrine liposomes, VAP-modified targeting coumarin liposomes, PEI-modified targeting coumarin liposomes and multifunctional targeting coumarin plus tetrandrine liposomes for 12 h, respectively. The final concentrations of coumarin was 10 μM. After the spheroids were washed with PBS for 3 times, the samples were scanned at the different layers from the top of the spheroids to the middle using a confocal laser scanning fluorescent microscope.

To evaluate the destruction effects of the varying formulations, GSCs spheroids were collected into 6-well culture plates, followed by being treated with vinorelbine liposomes, vinorelbine plus tetrandrine liposomes, VAP-modified targeting vinorelbine liposomes, PEI-modified targeting vinorelbine liposomes and multifunctional targeting vinorelbine plus tetrandrine liposomes at a concentration of 0.5 μM vinorelbine, respectively. Culture medium was used as blank control. After incubation for 48 h, the spheroids were fixed by 2.5% glutaraldehyde for 120 min, washed 3 times with cold PBS, and then dehydrated and embedded. The specimens were observed under a scanning electron microscope (JSM-5600 LV, JEOL, Japan).

### *In vivo* imaging observation

ICR mice (16–18 g) were obtained from Liao Ning Chang Sheng Biotechnology Co., Ltd. (Benxi, China). All procedures were performed according to guidelines of the Institutional Authority for Laboratory Animal Care of Liaoning University of Traditional Chinese Medicine. Glioma-bearing mice were prepared as our previous reports [45]. To evaluate the tumor accumulation ability in glioma-bearing mice, the noninvasive optical imaging systems were used. After inoculated for 14 days, the mice were randomly divided into seven groups (2 mice/group) and administered via tail vein injection with saline, free DiR, DiR liposomes, DiR plus tetrandrine liposomes, VAP-modified DiR liposomes, PEI-modified DiR liposomes and multifunctional targeting DiR plus tetrandrine liposomes (1 μg DiR each). Then, the mice were scanned at 1, 3, 6, 9, 12, 24 and 48 h using a Kodak multimodal imaging system (Fx Pro, NY, USA). The mice were sacrificed at 48 h, and the major organs, including heart, liver, spleen, lung, kidney and brain were removed immediately, and the fluorescence signal intensities in different tissues were photographed subsequently.

### Antitumor effects in glioma-bearing mice

Glioma-bearing mice were prepared as above. At day 10 after inoculation, the mice were randomly divided into 6 groups (15 mice/group) and treated with saline, vinorelbine liposomes, vinorelbine plus tetrandrine liposomes, VAP-modified targeting vinorelbine liposomes, PEI-modified targeting vinorelbine liposomes and multifunctional targeting vinorelbine plus tetrandrine liposomes via tail vein injection at a dose of 0.5 mg/kg vinorelbine, respectively. Administration was carried out every 2 days for consecutive 4 times. At day 25 after inoculation, 3 mice were anesthetized and the brains were assessed by magnetic resonance imaging (MRI, Siemens, Munich, Germany). 3 mice of each group were chosen to evaluate the preliminary toxicity. Blood samples (20 μL) were collected from the retro-orbital sinus and blood counts were obtained using an MEK-6318K Hematology Analyzer (Nihon Kohden, Japan). 3 mice of each group were sacrificed by cervical dislocation, the brain tissues were carefully isolated, and then brain slices were prepared via frozen section technique (5 μm). To observe the inhibiting effects to tumor cells after treatments with varying formulations *in vivo*, the Nissl staining assay was performed. The immunohistochemical staining for CD133 on brain glioma was carried out with antibodies for CD133 (1:300 dilution; Boster, Beijing, China). Immunohistochemical reactions were visualized using horseradish peroxidase, and the nuclei were counterstained with hematoxylin. The remaining 6 mice in each group were used for monitoring survival. The weights, behaviors and survivals of these mice were observed and Kaplane Meier survival curves were plotted for each group [50].

### The specificity to glioma sites

Coumarin was used as a fluorescent probe to evaluate the specificity of the multifunctional targeting liposomes to glioma sites. Briefly, 18 glioma-bearing mice were inoculated with GSCs as above. After inoculated for 14 days, the glioma-bearing mice were administered with saline, free coumarin, coumarin liposomes, VAP-mediated targeting coumarin liposomes, PEI-mediated targeting coumarin liposomes and multifunctional targeting coumarin plus tetrandrine liposomes via tail vein injection at a concentration of 50 μg coumarin each mice, respectively. After administration for 4 h, mice were sacrificed, and then hearts were perfused with 20 mL PBS for removing the blood in brains. Brains were removed to make cryosections (10 μm in thickness). The slices were stained with Hoechst 33258 (2 μg/mL) for 20 min in the dark and then washed 3 times with cold PBS. The samples were analyzed using a confocal laser scanning fluorescent microscopy [51].

### Statistical analysis

Data are presented as the means ± SD. ANOVA was used to determine the significance among groups and post hoc tests were used for multiple comparisons between individual groups. A value of *P* < 0.05 was considered to be significant.

## Supplementary Materials FIGURES AND TABLE


