# Supplementary material for: Multifunctional targeting vinorelbine plus tetrandrine liposomes for treating brain glioma along with eliminating glioma stem cells
Source: Oncotarget. 2016 Mar 25;7(17):24604–22. doi: 10.18632/oncotarget.8360 (PMC5029727; doi:10.18632/oncotarget.8360)
Supplement: Supplementary file 1 [file oncotarget-07-24604-s001.pdf]

Multifunctional targeting vinorelbine plus tetrandrine liposomes for treating brain glioma along with eliminating glioma stem cells

Supplementary Materials

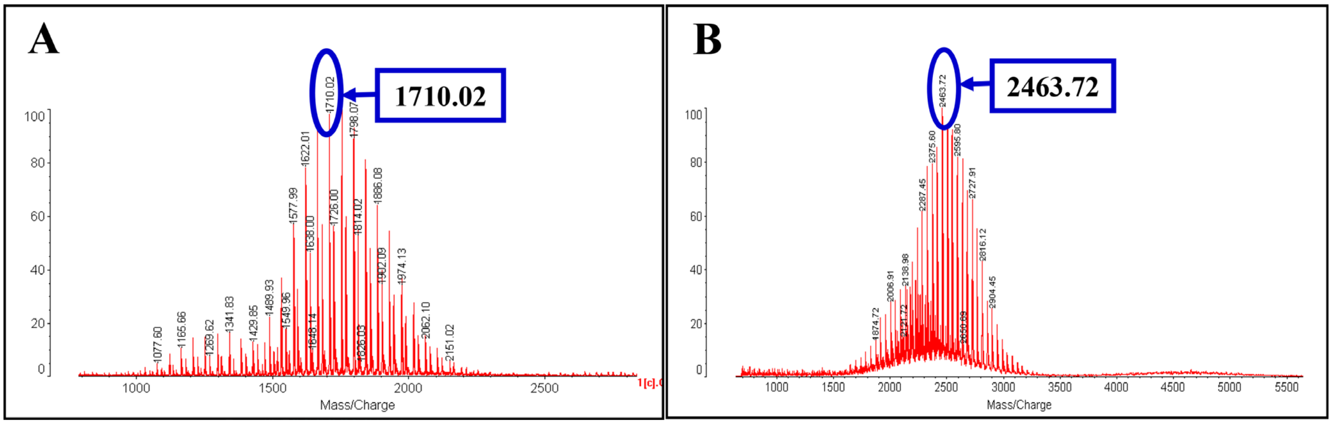

Supplementary Figure S1: MALDI-TOF-MS spectra of raw materials. Notes: (**A**) MALDI-TOF-MS spectra of TPGS1000. (**B**) MALDI-TOF-MS spectra of CHOL-PEG2000-NHS.

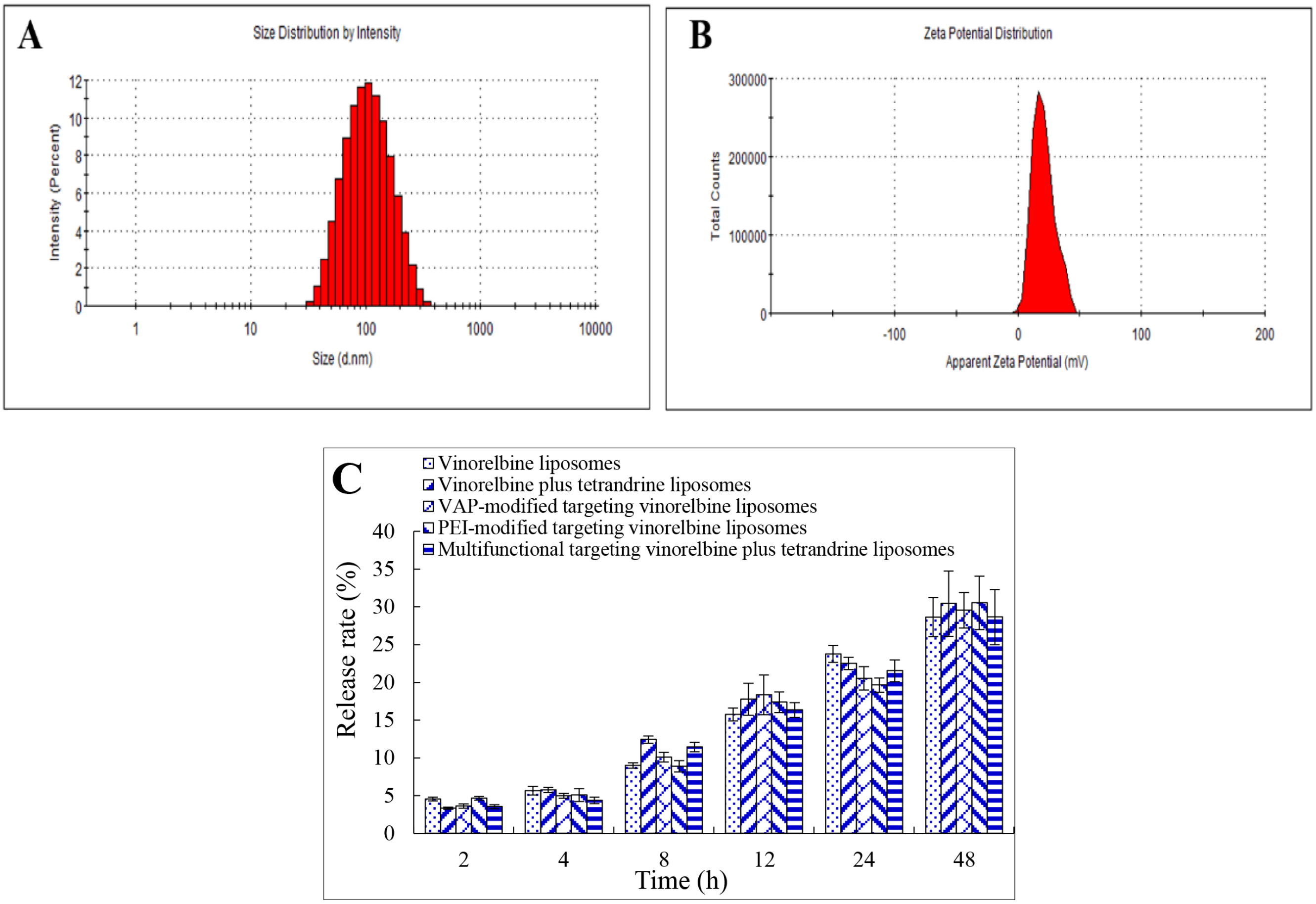

Supplementary Figure S2: Particle size distribution, zeta potential and drug release of multifunctional targeting tetrandrine plus vinorelbine liposomes. Notes: (**A**) Particle size of multifunctional targeting tetrandrine plus vinorelbine liposomes; (**B**) Zeta potential of multifunctional targeting tetrandrine plus vinorelbine liposomes; (**C**) Release rate of vinorelbine.

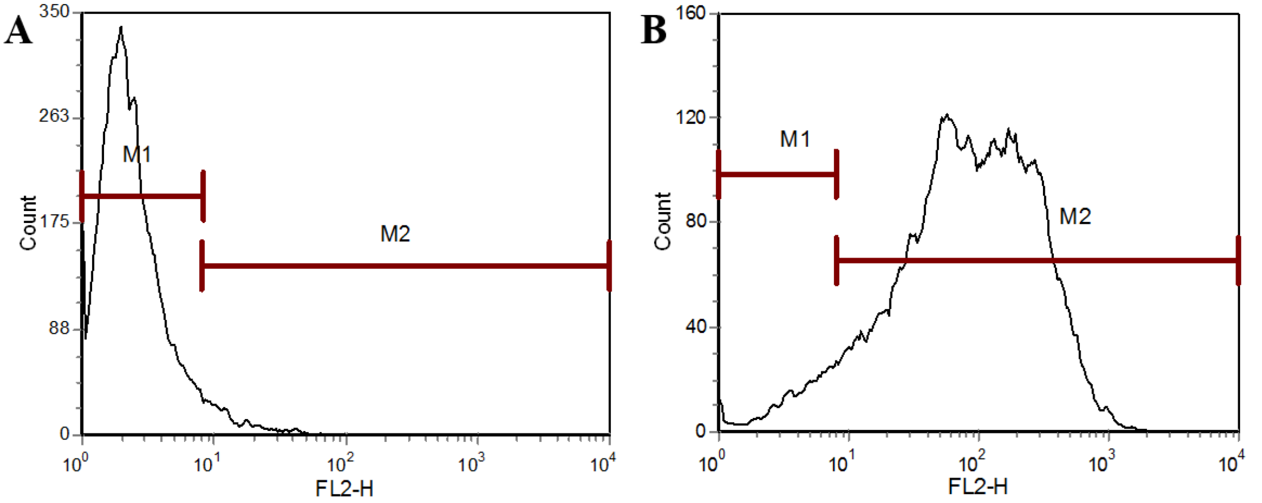

(A)

Histogram #	Low bound	High bound	# of Events	% of gated cells	Geometric Mean
1	0	8	9835	94.55	2.24
1	8	10000	597	5.74	14.18

(B)

Histogram #	Low bound	High bound	# of Events	% of gated cells	Geometric Mean
1	0	8	686	6.61	3.86
1	8	10000	9692	93.39	91.32

Supplementary Figure S3: Identification of GSCs. Notes: (**A**) GSCs treated as isotype controls; (**B**) GSCs stained with anti-mouse/rat nestin-phycoerythrin antibiodies.

Supplementary Table S1: Blood examination of the glioma-bearing mice after the administration of varying formulations

Assay	1	2	3	4	5	6
**WBC**[a]	6.76 ± 2.95	9.67 ± 1.69	8.07 ± 1.54	6.47 ± 0.96	6.73 ± 1.44	6.37 ± 2.54
**RBC**[b]	8.84 ± 0.72	8.25 ± 0.89	7.90 ± 0.63	7.82 ± 0.29	7.66 ± 0.52	7.62 ± 0.61
**HGB**[c]	151 ± 9.5	146 ± 6.6	131 ± 13.9	137 ± 5.3	127 ± 16.6	135 ± 13.1
**HCT**[d]	43.80 ± 2.77	42.07 ± 2.57	38.97 ± 3.35	40.00 ± 1.87	39.93 ± 1.98	38.9 ± 3.26
**MCV**[e]	49.63 ± 0.924	51.03 ± 1.42	49.30 ± 0.26	51.13 ± 0.55	49.53 ± 0.76	51.03 ± 1.46
**MCH**[f]	17.10 ± 0.62	17.73 ± 0.49	16.53 ± 0.45	17.53 ± 0.23	16.57 ± 0.25	17.70 ± 0.53
**MCHC**[g]	344.67 ± 8.74	347.33 ± 7.37	335.67 ± 7.51	342.67 ± 4.04	334.67 ± 4.58	347.00 ± 4.36
**RDW**[h]	12.73 ± 0.40	13.63 ± 0.21	13.57 ± 0.36	14.27 ± 0.65	13.19 ± 0.17	13.53 ± 0.13
**PLT**[i]	510.67 ± 24.50	530.00 ± 69.22	537.33 ± 25.54	495.00 ± 53.77	472.67 ± 29.02	457.33 ± 116.35
**PCT**[j]	0.19 ± 0.01	0.18 ± 0.21	0.19 ± 0.06	0.19 ± 0.03	0.17 ± 0.02	0.17 ± 0.05
**MPV**[k]	3.77 ± 0.06	3.77 ± 0.06	3.76 ± 5.44	3.83 ± 0.35	3.73 ± 0.58	3.87 ± 0.15
**PDW**[l]	14.33 ± 0.38	13.63 ± 0.25	13.77 ± 0.23	13.97 ± 0.76	14.43 ± 0.50	14.27 ± 0.04
**LYM**[m]	9.37 ± 2.23	6.93 ± 2.59	6.97 ± 1.13	7.33 ± 0.75	5.87 ± 0.80	6.17 ± 1.91
**MID**[n]	0.63 ± 0.06	0.63 ± 0.06	0.61 ± 0.17	0.43 ± 0.58	0.57 ± 0.05	0.47 ± 0.29
**GRN**[o]	2.80 ± 0.82	3.10 ± 1.5	1.57 ± 0.31	1.93 ± 0.31	1.83 ± 0.67	1.73 ± 0.57

Notes: Data are presented as the mean ± SD (n = 3). 1. Blank control; 2. Vinorelbine liposomes; 3. Vinorelbine plus tetrandrine liposomes; 4. VAP-modified targeting vinorelbine liposomes; 5. PEI-modified targeting vinorelbine liposomes; 6. Multifunctional targeting vinorelbine plus tetrandrine liposomes. (a) white blood cells; b, red blood cells; c, hemoglobin; d, hematocrit; e, mean corpuscular volume; f, mean corpuscular hemoglobin; g, mean corpuscular hemoglobin concentration; h, red cell distribution width; i, platelets; j, plateletcrit; k, mean platelet volume; l, platelet distribution width; m, lymphocyte; n, intermediate cell; o, neutrophil granulocyte.
